# Correspondence

**Published:** 1900-07

**Authors:** S. B. Little


### *CORRESPONDENCE.*

---

FIVE FORKS, GA., June 21, 1900.

*Editor Atlanta Journal-Record of Medicine, Atlanta, Ga.:*

DEAR SIR—I have a case to report which I think will interest some of THE JOURNAL readers.

Was called November 28, 1899, to see John C. Coe, aged 40, a laborer. He was running an engine at a saw-mill and gin. He shut off the steam to stop the gin, and, without waiting for the machinery to stop, he grabbed a large belt running over a wheel and pulled the belt off. In stooping, a set screw in a pulley under him caught in an old croker-sack apron he had on, wound up the apron, and with it his scrotum, snatching off apron, scrotum and all. When I reached him he was lying on the floor covered with a quilt. I took off the quilt and found his testes entirely denuded down to the visceral tunica, or deep tunic, exposing spermatic cord, pudic and epigastric arteries (the mouths of them); his penis was also entirely denuded, leaving it looking like a long red eel skinned. Now what was I to do? Of course I stopped the hemorrhage first, and gave hypo of morphine and strychnine for pain and shock. The pulley in going over had carried him over also for several rounds, bruising him badly. If I should remove his testes I had no skin or anything but muscle in six or eight inches of his testes, besides, he begged to be allowed to keep them as long as he lived, and so did his wife. I thought it a case of death, probably, any way, so dressed him well, using vitogen and bismuth as an antiseptic and germicide, besides, this, I found, did not cause the pain on raw surfaces that iodoform did. I returned the next day and found him resting well—wanted to sit up and drink buttermilk. I continued to carry out this treatment, and at the end of one week he could roll his seeds about in his hands like hickorynuts, there being a tough membrane formed over them. His penis had a nice, tender, new skin formed over it. He continued to improve until I began to look about for a bull's bag for him to carry his tools in.

One night he got mad with his wife, rolled and tumbled over his bed, got a quilt between his knees and rubbed all the new, tender skin off of his penis, got hold of a gallon of buttermilk, drank it, and died the next day.

Did I treat this case right? or should I have done some surgery anyway against his request? Yours truly,

S. B. LITTLE, M.D.

